# Preoperative carbohydrate loading and intraoperative goal-directed fluid therapy for elderly patients undergoing open gastrointestinal surgery: a prospective randomized controlled trial

**DOI:** 10.1186/s12871-021-01377-8

**Published:** 2021-05-21

**Authors:** Xia Liu, Peng Zhang, Meng Xue Liu, Jun Li Ma, Xin Chuan Wei, Dan Fan

**Affiliations:** 1 Department of Anesthesiology, Sichuan Academy of Medical Sciences and Sichuan Provincial Peoples Hospital, No. 32 West Second Section, First Ring Road, Chengdu, Sichuan China; 2 North Sichuan Medical College, Nanchong, Sichuan China

**Keywords:** Carbohydrate, Goal-directed fluid therapy, Elderly, Gastrointestinal surgery, Outcome

## Abstract

**Background:**

The effect of a combination of a goal-directed fluid protocol and preoperative carbohydrate loading on postoperative complications in elderly patients still remains unknown. Therefore, we designed this trial to evaluate the relative impact of preoperative carbohydrate loading and intraoperative goal-directed fluid therapy versus conventional fluid therapy (CFT) on clinical outcomes in elderly patients following gastrointestinal surgery.

**Methods:**

This prospective randomized controlled trial with 120 patients over 65years undergoing gastrointestinal surgery were randomized into a CFT group (*n*=60) with traditional methods of fasting and water-deprivation, and a GDFT group (*n*=60) with carbohydrate (200ml) loading 2h before surgery. The CFT group underwent routine monitoring during surgery, however, the GDFT group was conducted by a Vigileo/FloTrac monitor with cardiac index (CI), stroke volume variation (SVV), and mean arterial pressure (MAP). For all patients, demographic data, intraoperative parameters and postoperative outcomes were recorded.

**Results:**

Patients in the GDFT group received significantly less crystalloids fluid (1111442.9ml vs 1411412.6ml; *p*<0.001) and produced significantly less urine output (200ml [150300] vs 400ml [290500]; *p*<0.001) as compared to the CFT group. Moreover, GDFT was associated with a shorter average time to first flatus (5614.1h vs 6422.3h; *p*=0.002) and oral intake (7216.9h vs 8526.8h; *p*=0.011), as well as a reduction in the rate of postoperative complications (15 (25.0%) vs 29 (48.3%) patients; *p*=0.013). However, postoperative hospitalization or hospitalization expenses were similar between groups (*p*>0.05).

**Conclusions:**

Focused on elderly patients undergoing open gastrointestinal surgery, we found perioperative fluid optimisation may be associated with improvement of bowel function and a lower incidence of postoperative complications.

**Trial registration:**

ChiCTR, ChiCTR1800018227. Registered 6 September 2018 - Retrospectively registered.

**Supplementary Information:**

The online version contains supplementary material available at 10.1186/s12871-021-01377-8.

## Background

Patients 65years old are termed elderly [1]. Elderly patients typically present with a range of physical, pharmacological, and psychological comorbidities that must be carefully considered by clinicians and anesthesiologists prior to any major surgical operation [2]. Indeed, more advanced age remains a key risk factor associated with higher rates of postoperative morbidity and mortality [3].

Elderly patients are more susceptible to deleterious effects such as dehydration, hypovolemia and hemodynamic instability induced by prolonged fasting [4]. Intraoperative hypovolemia can, in turn, result in a range of serious postoperative complications including hypotension and severe arrhythmia, whereas hypervolemia can also cause serious problems such as anastomotic leaks, infection, pulmonary edema, or even death [5,10]. As such, it is clear that both insufficient and excess fluid infusion can cause harm in patients [11]. Owing to age-related declines in organ function and greater difficulty adjusting fluid preloading, elderly patients are thus at a substantially higher risk of postoperative mortality.

Preoperative fluid therapy mainly aims to prevent the patients from a dehydrated or hypovolemic state before anesthesia induction. Therefore, multiple ERAS (Enhanced Recovery After Surgery) guidelines include the oral intake of carbohydrate loading (200ml) 2h before surgery, which may help to decrease postoperative complications, such as postoperative nausea and vomiting and wound infection. Besides, the use of routine hemodynamic measurements (such as arterial blood pressure or heart rate) is often a relatively imprecise means of monitoring for changes in blood volume [12, 13]. In contrast, goal-directed fluid therapy approaches rely upon monitoring more advanced hemodynamic variables such as SVV and pulse pressure variability, which are more sensitive to hypovolemia and therefore allow for optimal preloading by enabling clinicians to appropriate titrate fluids and inotropic substances [14, 15].

GDFT has been shown to reduce the duration of hospitalization and to decrease the incidence of postoperative complications by 2050% in previous systematic reviews [16,18]. However, there have been few studies regarding whether preoperative carbohydrate loading combined with intraoperative GDFT is similarly beneficial in elderly patient populations. As such, in the present study we conducted fluid optimisation in an elderly patient population via the use of hemodynamic indicators (CI, SVV, MAP) and vasoactive drugs, as necessary to. We hypothesized that preoperative carbohydrate (200ml) loading and intraoperative GDFT based upon SVV, CI and MAP may lower postoperative hospitalization and postoperative complications in elderly patients undergoing gastrointestinal surgery.

## Methods

### Patients

This prospective randomized trial was performed at the Sichuan Provincial Peoples Hospital (Chengdu, China). The trial was registered in the center of Chinese Clinical Trial Registry (ChiCTR1800018227). After the Ethics Committees of the Sichuan Academy of Medical Sciences and Sichuan Provincial Peoples Hospital approved this study (Approval Number 2018157), we enrolled patients between May 2018 and November 2019 to receive two different protocols for perioperative fluid therapy. Eligible patients were individuals >65years old that were scheduled to undergo elective open gastrointestinal surgery (anticipated operating time>2h) and that matched the ASA (American Society of Anesthesiologists) class 24 criteria. Prior studies have shown that the accuracy of Vigileo/FloTrac is reduced in patients with abnormal sinus rhythm or intraabdominal pressure values [19], and as such patients were excluded from this study if they had no sinus rhythm, or if they had a history of gastrointestinal surgery, peripheral artery disease, or with high risk of reflux and aspiration (pyloric obstruction or achalasia of the cardia). All patients provided written informed consent before participation in the trial.

### Randomization and blinding

A series of random numbers generated by SPSS software were used to randomize the grouping of patients. The patients numbered 1120 according to the time of elective surgery were randomized either to the GDFT group or the CFT group. The blinding of group assignment was ensured by the opaque envelopes. The random serial numbers and the envelopes were kept by an investigator who was not involved in the conduct of the trial. Subjects, surgeons, patients themselves, ICU physicians and professionals for data recording, collection and analysis were blind to group assignment. However, the attending anesthesiologist and investigators could not be blinded because of the existence of cardiac index trending monitor in the GDFT group.

### Intraoperative management

All patients were monitored for pulse oximetry, five-lead-electrocardiogram, blood pressure, heart rate, temperature and end-tidal carbon dioxide concentrations. Central venous pressure (CVP) monitoring was conducted in some patients as decided by the attending anesthesiologist based on the patients physical situation and surgical needs after the surgery. Patients in both groups received a minimum of one peripheral intravenous access (18G), with a central venous catheter being inserted via the internal jugular vein and with radial arterial catheters being introduced via the non-dominant forearm under low-dose dexmedetomidine for sedation in the operating room at 7:30AM on the day of surgery. For each patient, the following was recorded: baseline characteristics, surgery type, and basic hemodynamics variables (blood pressure, MAP, HR). An initial arterial blood gas analysis was also conducted at this time. For patients in the GDFT group, the radial arterial lines were attached to the fourth generation Vigileo/FloTrac monitor (Edwards Life Sciences, CA, USA). The technique of point-of-care gastric ultrasound was performed to make sure that the patient was not in high risk of reflux aspiration on account of carbohydrate loading 2h before the gastrointestinal operation. Anesthesia was then induced by using propofol (22.5mg.kg^1^) or etomidate (0.2mg.kg^1^) in combination with sufentanil (1525 g) and cisatracurium (0.150.2mg.kg^1^). Anesthesia was maintained with remifentanil (0.20.25 g.kg^1^min^1^), dexmedetomidine (0.40.7 g.kg^1^h^1^) and sevoflurane at a MAC (minimum alveolar concentration) of 11.3 in order to achieve a BIS (bispectral index) value of 4060. Each patient was ventilated with a tidal volume of 8ml/kg without PEEP (positive end-expiratory pressure) at a respiratory rate sufficient to achieve an end-tidal carbon dioxide value of 3545mmHg. Flow rates were set to 23L/min, and 60% oxygen was administered for the operative duration.

### Study protocol

**Fig. 1:**
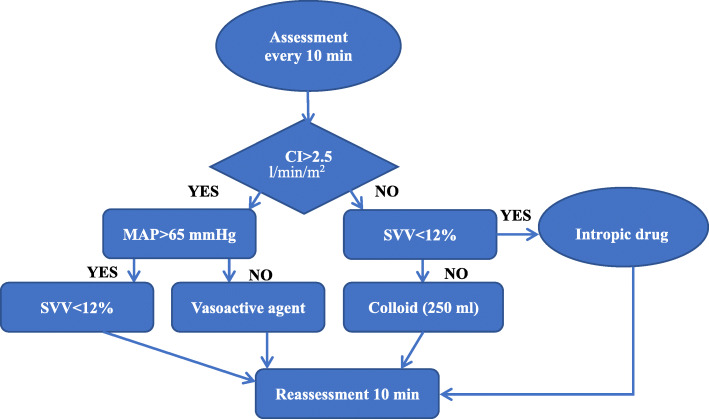
Flow chart of the GDFT

The bowel preparation was performed by the surgeons instruction 1day before surgery. The GDFT group was fasted for 8h and drunk 200ml carbohydrate (Shuneng; Yi Chang Ren Fu Pharmaceutical Co Ltd., China) 2h before the operation. The CFT group was fasted for 8h and prohibited drinking for 4h before surgery. The CFT group was initially infused with a balanced crystalloid solution at 2ml/kg/h for baseline fluid therapy and 6ml/kg/h during surgery (from incision to suturing). In addition, additional bolus (crystalloid or colloids) could be infused at the discretion of the attending anesthesiologist, or vasoactive drugs could be administered to ensure that routine monitoring variables remained within the normal range (heart rate<100bpm, MAP >65mmHg, urine output >0.5ml/kg/h). Patients in the GDFT treatment group were infused based on the Vigileo/FloTrac instrument. If CI was 2.5l/min/m^2^, MAP was >65mmHg, and SVV was <12%, the administration rate was maintained at 6ml/kg/h for the entire surgical operation. When CI reached 2.5l/min/m^2^ with SVV>12%, additional colloid 250ml boluses were administered within 10min. When CI was 2.5l/min/m^2^ and SVV was <12%, dobutamine was injected to achieve a CI of 2.5l/min/m^2^. Ephedrine boluses of 318mg or norepinephrine infusions were administered when CI was 2.5l/min/m^2^ and MAP was <65mmHg (Fig.1). All hemodynamic variables were reassessed after 10min.

### Data collection

We recorded lactate values at 5min prior to the induction of anesthesia (T1), 60min after the operation was initiated (T2), and at the end of surgery (T3). All intraoperative data were collected upon the completion of the operation, including operative duration, duration of anesthesia, types and amounts of fluid infusion, estimated blood loss, urine output, and vasoactive agent use. Following intensive care unit (ICU) or ward admission, the management of all patients was conducted by medical staff blinded to patient group assignments. Time to first flatus and time to first oral intake was recorded. Following patient discharge, we recorded the postoperative hospital stay, amount of hospital expenses, admission to ICU, postoperative complications and mortality. The predefined criteria for complications in the study protocol were described in Additional file 1.

### Statistical analysis

The sample size calculation was based on a study by Wackeling et al. [20], which showed the postoperative complication rate was 59.3% in patients receiving standard of care fluid therapy as compared to 37.5% in the GDFT group. For a reduction in the rate of postoperative complication from 60 to 32% in the GDFT group with type I error of 0.05 and a power of 80%, a sample size of 50 patients was calculated by our researchers for each group. Owing to loss of about 20% of patients allocated to groups as a result of changes in scheduled surgical procedure, our study finally included 60 patients per group. SPSS 26.0 (IBM, IL, USA) was used for all statistical testing. Data were given as meanSD, absolute values (percentages) or medians (25th75th percentiles). Data were compared via Students t-tests (for normally distributed variables), Wilcoxon rank-sum tests (for non-normally distributed variables), and chi-squared or Fishers exact tests (for categorical variables). Normality was assessed via the Kolmogorov-Smirnov test. Arterial blood lactate concentrations were normally compared via analysis of variance (ANOVA) on repeated measurements with Bonferroni correction against baseline, whereas they were compared via the Friedman test when non-normally distributed. *p*<0.05 was the significance threshold.

## Results

**Fig. 2:**
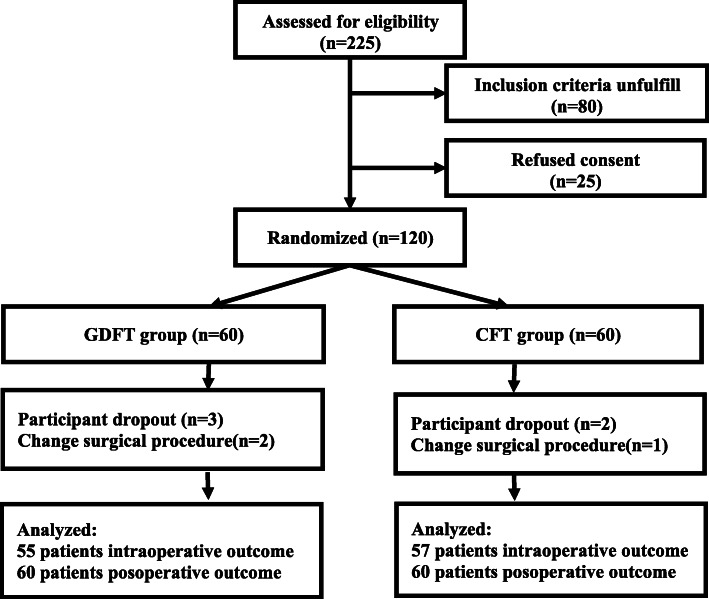
Trial flow chart

In total, 225 patients were eligible for inclusion in the present study between May 2018 and November 2019. After excluding patients based on our exclusion criteria and failure to give consent, the remaining 120 patients were randomized into the GDFT (*n*=60) and CFT (*n*=60) groups. Among these patients, 5 (3 and 2 in the GDFT and CFT groups, respectively) dropped out of the study, 3 (2 and 1 in the GDFT and CFT groups, respectively) were found to be inoperable (Fig.2). In total, our final analyses thus included 55 patients in the GDFT group and 57 patients in the CFT group.

There were no significant differences between groups with respect to demographic characteristics, surgical category, or ASA classification (*p*>0.05). Operative duration and duration of anesthesia were also similar between groups. Intraoperative blood loss was comparable between groups. Patients in the CFT group were administered a significantly larger volume of fluids relative to patients in the GDFT group (2033468.7ml vs 1697536.9ml; *p*<0.01), with the difference primarily being in the form of crystalloid infusion (1411412.6ml vs 1111442.9ml; *p*<0.001). Urine output was also significantly greater in the CFT group compared with the GDFT group (400ml [290500] vs 200ml [150300]; *p<0.001*). There were no significant differences between groups with respect to intraoperative colloid infusion volume. With respect to vasoactive drugs, dobutamine was used more frequently in the GDFT group. There was also a significant increase in arterial lactate concentration in the CFT group relative to baseline at the end of surgery (1.0mmol/l (0.81.2) vs 0.7mmol/l (0.60.95); *p*<0.001). No other differences with respect to lactate values were detected between these two groups. For full details regarding these findings, see Tables1 and 2.

**Table 1:** Patient baseline characteristics

	GDFT group (*n*=55)	CFT group (*n*=57)
Age (years)	68.35.49	69.26.55
Height (m)	1.610.07	1.620.08
Weight (kg)	60.2010.53	59.2610.62
BMI (kg/m^2^)	23.022.75	22.343.11
Sex (M/F)	36/19	40/17
ASA II/III/IV	38/12/5	40/10/7
Type of surgery		
Gastrectomy	16 (29.1%)	15 (26.3%)
Colectomy	20 (36.4%)	25 (43.9%)
Proctectomy	19 (34.5%)	17 (29.8%)

*Note*: Values are absolute numbers (percentages) or means SD (standard deviation)

*GDFT* goal-directed fluid therapy, *CTF* conventional fluid therapy, *BMI* body mass index, *ASA* American Society of Anesthesiologists

**Table 2:** Comparison of intraoperative outcomes and blood lactate concentrations

	GDFT group (*n*=55)	CFT group (*n*=57)	*P*
Duration of anesthesia (min)	25464.8	24460.6	0.413
Duration of surgery (min)	20859.9	20057.1	0.469
Estimated blood loss (ml)	200 (150300)	200 (100400)	0.773
Total intraoperative fluids (ml)	1697536.9	2033468.7	0.001^##^
Crystalloid (ml)	1111442.9	1411412.6	0.000^###^
Colloid (ml)	623211.9	586216.3	0.370
Urine output (ml)	200 (150300)	400 (290500)	0.000^###^
Intraoperative vasoactive drugs			
Ephedrine	25	31	0.345
Norepinephrine	10	4	0.091
Dobutamine	9	2	0.022^#^
Nitroglycerin	2	3	1.000
Lactate (mmol/l)			
T1	0.8 (0.61.3)	0.7 (0.60.95)	0.363
T2	0.9 (0.61.3)	0.8 (0.651.1)	0.243
T3	0.9 (0.71.3)	1.0 (0.81.2) ^***^	0.752

*Note*: Values are absolute number, meanSD (standard deviation) or median (2575 percentage); *** *p*<0.001 analysis of variance (ANOVA) on repeated measurements with Bonferroni correction against baseline; ^#^ *p*<0.05, ^##^ *p*<0.01, ^###^ *p*<0.001 analysis of variance by GDFT groups vs CFT group (T-test; Wilcoxon rank-sum tests, or Fishers exact tests); *T1* 5min prior to induction of anesthesia, *T2* 60min after the initiation of surgery, *T3* at the end of surgery

An intention-to-treat analysis and per protocol analysis for postoperative outcomes were presented in Table3. With respect to postoperative bowel function, we found that GDFT was associated with a shorter time to first flatus (5614.1h vs 6422.3h; *p*=0.002) and to first oral intake (7216.9h vs 8526.8h; *p*=0.011) relative to the CFT group. In addition, there were significantly fewer instances of postoperative complications among patients in the GDFT group relative to the CFT group (15 (25.0%) vs 29 (48.3%) patients; *p*=0.013). Further analysis of predefined types of complications found that there was a lower rate of infection complications in in the GDFT group relative to the CFT group (5 (9.1%) vs 13 (22.8%) patients; *p*=0.048). However, GDFT was not associated with any significant differences in postoperative hospitalization duration (9.12.8days vs 9.73.2days; *p*=0.290), ICU admission (6 (10.0%) vs 7 (11.7%) patients; *p*=0.769), or hospitalization expenses (57,59916,363 RMB vs 62,80219,891 RMB; *p*=0.120). In the per protocol analysis, the GDFT group was also associated with a lower rate of complications (14 (25.5%) vs 27(47.4%) patients; *p*=0.016) relative to the CFT group. Again, there was no difference in hospital stay (9.22.9days vs 9.73.1days; *p*=0.397) and hospitalization expenses (56,84516,446 RMB vs 60,57116,579 RMB; *p*=0.235). Although there were no significant differences between groups with respect to postoperative mortality (0 (0%) vs 2 (3.3%) patients; *p*=0.496), two patients in the CFT group did die on postoperative day 3 as a result of acute massive gastrointestinal hemorrhage and acute myocardial infraction respectively.

**Table 3:** Comparison of postoperative outcomes and complications

	GDFT group	CFT group	*p*
Number of patients			
ITT analysis	60	60	
Per protocol analysis	55	57	
Primary outcome			
Patients with complications			
ITT analysis	15 (25.0%)	29 (48.3%)	0.013
Per protocol analysis	14 (25.5%)	27 (47.4%)	0.016
List of complications (Per protocol analysis only)			
Infection	5 (9.1%)	13 (22.8%)	0.048
Respiratory	5 (9.1%)	9 (15.8%)	0.284
Cardiovascular	6 (10.9%)	9 (15.8%)	0.448
Abdominal	6 (10.9%)	10 (17.5%)	0.316
Renal	1 (1.8%)	2 (3.5%)	1.000
Others	2 (3.6%)	2 (3.5%)	1.000
Secondary outcomes			
Time to first flatus (hours)			
ITT analysis	5614.1	6422.3	0.002
Per protocol analysis	5513.9	6522.6	0.004
Time to first oral taken (hours)			
ITT analysis	7216.9	8526.8	0.011
Per protocol analysis	7217.4	8527.5	0.002
Postoperative hospitalization (days)			
ITT analysis	9.12.8	9.73.2	0.290
Per protocol analysis	9.22.9	9.73.1	0.397
Amount hospital charges (RMB)			
ITT analysis	57,59916,363	62,80219,891	0.120
Per protocol analysis	56,84516,446	60,57116,579	0.235
Admission to ICU			
ITT analysis	6 (10%)	7 (11.7%)	0.769
Per protocol analysis	5 (9.1%)	6 (10.5%)	0.799
Postoperative mortality			
ITT analysis	0 (0%)	2 (3.5%)	0.496
Per protocol analysis	0 (0%)	2 (3.3%)	0.496

*Note*: Values are absolute (percentage) or meanSD (standard deviation)

*ITT* intention to treat analysis, *Per protocol* only patients performed intraoperative protocol in full extent, *RMB* Renminbi, *ICU* intensive care unit

## Discussion

In this prospective randomized study, we evaluated the effect of using a standard of care intraoperative fluid therapy as compared to an intraoperative goal-directed fluid therapy combined with a preoperative carbohydrate loading on the incidence of postoperative complications in elderly patients undergoing open gastrointestinal surgery. We observed a significantly faster restoration in bowel function and lower rate of postoperative complications in the GDFT group as compared to the standard of care group.

The primary outcome was initially the length of hospitalization. The reduction in the hospital stay of 1 day was used for simple size calculation. However, during the pilot experiments, we noted that the improvement in the length of hospitalization was lower than that we had been predicted in the original study design and the study may not be finished with the power originally planned. We found that hospitalization duration can be influenced by a range of factors such as patients wishes, preoperative physical condition, health care system requirements, and institution-specific differences in treatment regimens. All of these factors thus have the potential to influence the relationship between GDFT and postoperative hospitalization duration. However, we found the interventions may have more effect on the rate of postoperative complications than the length of hospitalization. Therefore, we finally determined the rate of postoperative complications as the primary outcome and the length of hospitalization as one of the secondary outcomes.

For this study, we opted to utilize CI rather than the oxygen delivery index as the key target variable for our GDFT protocol as it can be readily and continuously monitored via radial artery pulse waveform analysis. When arterial oxygen saturation and hemoglobin levels are adequate, CI can serve as an effective measurement to evaluate oxygen supply within tissues and organs [21]. As the use of CI and SVV with the Vigileo/FloTrac monitor has been found to be potentially unreliable in patients with irregular heart rhythms [19, 22] and in patients with poorly controlled intraoperative ventilation [23], we did not include patients without in sinus rhythm in the present study, and all patients were ventilated using a tidal volume of 8ml/kg.

Excess fluid administration can result in a range of problems including increased rates of postoperative cardiac morbidity, pneumonia, respiratory failure, delayed wound healing, and anastomotic leak as a consequence of intestinal edema in patients undergoing colorectal surgery [8]. As shown in some prior trials [21, 24, 25], our study also found that the GDFT protocol is associated with reductions in the rate of postoperative complications. However, the rates of postoperative complications in some previous studies were higher than in our present analysis. There are two potential reasons for this discrepancy. For one, Mayer et al. [21] selected high-risk patients with at least two risk factors according to risk index of Lee [26] as their experimental subjects. Furthermore, Benes et al. [25] recruited patients that had to meet both operation-related and patient-related high-risk criteria. In contrast, all patients we included were over the age of 65, some of whom may be in the low-to-moderate risk category. In addition, we only monitored the incidence of postoperative complications that occurred during hospitalization, whereas Benes et al. monitored patients for 30days [25]. Lastly, the lower incidence of postoperative complications in our study may also be related to preoperative oral carbohydrate loading, which may reduce the metabolic and inflammatory response after surgery and improved surgical clinical outcomes [27]. Together, our results suggest that perioperative fluid optimisation reduces the incidence of postoperative complications in elderly patients, rather than only in high-risk patients.

The traditional view considered that the preoperative 8-h food fasting and 4-h liquid fasting accompanied by a decrease in the gastric volume and acidity can reduce the risk of reflux aspiration and misabsorption [28]. However, a long time for preoperative fasting can result in harms to the body, such as loss of water and nutrients, instability of hemodynamics, delayed wound healing, the extension of hospital stays [29]. Therefore, ERAS (Enhanced Recovery After Surgery) guidelines suggested the oral intake of carbohydrate loading 2h before surgery [30]. In our study, we performed goal-directed fluid administration in combination with preoperative carbohydrate loading as compared to conventional clinical care. The preoperative oral carbohydrate loading combined with intraoperative ERAS may be worth popularizing in the clinical practice owing to their beneficial effect on postoperative outcomes.

Increased arterial lactate levels are closely linked to tissue hypoxia and blood volume insufficiency [31]. In the context of suboptimal fluid management, postoperative restoration of gastrointestinal motility and oral food intake may be delayed due to higher lactate levels. In our study, we did not observe any significant differences in arterial lactate values at analyzed time points between groups. However, we did find that lactate values in the CFT group increased at the end of surgery relative to baseline values. As a systematic review and meta-analysis demonstrated that perioperative GDFT facilitated gastrointestinal functional recovery such as shortening the first time to exhaust and the first time to take oral diet compared with CFT [32]. The slower recovery of bowel function observed in the CFT group may thus be associated with gastrointestinal mucosal hypoperfusion and increase in lactate production [33].

Intraoperative reduced urine output is an independent predictor of AKI (acute kidney injury) after major abdominal surgery [34]. In this study, we found that patients in the GDFT treatment group required reduced crystalloid administration and exhibited reduced urine output relative to patients in the CFT group. However, postoperative AKI occurred in one patient in the GDFT group and two patients in the CFT group. This outcome may be explained by work conducted by Kheterpal et al. [35], as these authors suggested that urine is unreliable when used as a marker of blood volume and renal function, given that it can be influenced by neurohormonal signaling in response to operative stress. The administration of diuretics and vasoactive agents has also been linked to the incidence of acute renal failure. Furthermore, Myles et al. [36] suggested that patients undergoing major abdominal surgery in the restrictive fluid group was associated with a significantly higher rate of postoperative AKI and was not associated with a significantly higher risk of renal-replacement therapy at 90days than those in the liberal fluid group. However, in our study, we found no statistically differences in the postoperative AKI between the CFT group and the GDFT group. Therefore, the intraoperative goal-directed fluid therapy appeared to be at lower risk of postoperative AKI than the restrictive regimen, which deserves more larger, higher-quality RCTs to further study [37].

There are multiple limitations to the present analysis. For one, in an effort to avoid potential compounds we did not utilize the CI and SVV trending monitor for patients in the CFT group, so we were not able to compare these parameters between groups. Moreover, we were not able to blind investigators intraoperatively to patient treatment strategies. Additionally, the professional level of the attending anesthesiologists and physicians for postoperative treatment in the ICU and hospital wards should be equivalent between groups. Otherwise, it is possible that higher rates of postoperative complications may result from poorer postoperative care in the CFT group. Moreover, although the two interventions including preoperative carbohydrate loading and intraoperative GDFT are the most efficient measures of the ERAS protocols to perform perioperative fluid optimization, it seems that we may not differentiate the beneficial effects between them on postoperative outcomes due to the trial design, which was worthy of further study. Lastly, we did not pre-define standardized discharge criteria in the present study, as discharge may be influenced by a range of factors including patient demands, health care system capacity, and specific treatment regimens used. Thus, the duration of postoperative hospitalization could be also biased.

## Conclusion

The results of this study indicate that preoperative carbohydrate loading and intraoperative fluid optimization guided by CI, SVV, and MAP may be associated with more rapid improvements in bowel function and a decreased incidence of postoperative complications in elderly patients undergoing open gastrointestinal surgery. Therefore, the combination of a goal-directed fluid protocol and preoperative carbohydrate loading can achieve some clinical benefits and is worth to be applied for elderly patients undergoing open gastrointestinal surgery.

## Supplementary Information

**Additional file 1.** Types and definitions of postoperativecomplications.

## Data Availability

All data generated or analyzed during this study are included in this published article and supporting data can be obtained from the corresponding author.

## Abbreviations

GDFT: Goal-directed fluid therapy
CTF: Conventional fluid therapy
BMI: Body mass index
ASA: American Society of Anesthesiologists
MAP: Mean arterial pressure
HR: Heart rate
RMB: Renminbi
ICU: Intensive care unit
AKI: Acute kidney injury
