# Supplementary material for: Preoperative carbohydrate loading and intraoperative goal-directed fluid therapy for elderly patients undergoing open gastrointestinal surgery: a prospective randomized controlled trial
Source: BMC Anesthesiol. 2021 May 21;21:157. doi: 10.1186/s12871-021-01377-8 (PMC8139051; doi:10.1186/s12871-021-01377-8)
Supplement: Supplementary file 1 — Additional file 1. Types and definitions of postoperativecomplications. [file 12871_2021_1377_MOESM1_ESM.docx]

Additional file 1: Types and definitions of complications.

| **Infection** |
| --- |
| Pneumonia: chest x-ray, WBC >12x10^3^ or < 4x10^3^ /l |
| Wound infection: clinical diagnosis |
| Intra-abdominal infection: abdominal CT |
| Urinary tract infection: urine analysis |
| Catheter-related infection: the tip of catheter culture and blood culture |
| **Respiratory** |
| Pulmonary embolism: CTPA |
| Atelectasis: chest CT |
| Pleural effusion: chest CT |
| Postoperative ventilator support: > 12 h |
| **Cardiovascular** |
| Hypotension: MAP < 50 mmHg |
| Arrhythmia: ≥ Lown II, ≥ 5/min atrial extrasystoles, AF, VF |
| Acute myocardial infraction: ECG ischemic signs, troponin T > 0.03 ng/ml |
| Acute heart failure: NT-pro BNP > 300 ng/l, chest x-ray (pulmonary edema), echocardiography |
| **Abdominal** |
| Bleeding: hematemesis or hematochezia, occult blood test, endoscopy procedure |
| Obstruction: absence of flatus, abdominal x-ray |
| Anastomotic leak: peritonitis, abdominal CT, |
| **Renal** |
| Acute kidney injury: Urine output < 0.5 ml//kg/h and the duration of oliguria > 6 h |
| Chronic renal dysfunction: creatinine > 133 umol/l, eGFR < 60 ml/min/1.73m^2^ |
| **Others** |
| Including other complications deemed as life disabling or threatening |

Note: WBC = white blood cell count; CT = computed tomography; CTPA = computed tomographic pulmonary angiography; MAP = mean arterial pressure; AF = atrial fibrillation; VF = ventricular fibrillation; ECG = electrocardiogram; eGFR = estimated glomerular filtration
